# Evaluation of Phytochemical and Antioxidant Properties of 15 Italian *Olea europaea* L. Cultivar Leaves

**DOI:** 10.3390/molecules24101998

**Published:** 2019-05-24

**Authors:** Francesca Nicolì, Carmine Negro, Marzia Vergine, Alessio Aprile, Eliana Nutricati, Erika Sabella, Antonio Miceli, Andrea Luvisi, Luigi De Bellis

**Affiliations:** Department of Biological and Environmental Sciences and Technologies, University of Salento, 73100 Lecce, Italy; francesca.nicoli@unisalento.it (F.N.); carmine.negro@unisalento.it (C.N.); alessio.aprile@unisalento.it (A.A.); eliana.nutricati@unisalento.it (E.N.); erika.sabella@unisalento.it (E.S.); antonio.miceli@unisalento.it (A.M.); andrea.luvisi@unisalento.it (A.L.); luigi.debellis@unisalento.it (L.D.B.)

**Keywords:** *Olea europaea* L., phenolic compound, antioxidant activity, by-product, HPLC ESI/MS-TOF

## Abstract

Olive leaf extracts are of special interest due to their proven therapeutic effects. However, they are still considered a by-product of the table olive and the oil industries. In order to learn possible ways of exploiting this waste for health purposes, we investigated the phytochemical profiles and antioxidant activities in the leaves of 15 Italian *Olea europaea* L. cultivars grown in the same pedoclimatic conditions. The phenolic profiles and amounts of their seven representative compounds were analyzed using HPLC ESI/MS-TOF. The antioxidant activities were determined using three different antioxidant assays (DPPH, ORAC, and superoxide anion scavenging assay). Wide ranges of total phenolic content (11.39–48.62 g GAE kg^−1^ dry weight) and antioxidant activities (DPPH values: 8.67–29.89 µmol TE mg^−1^ dry weight, ORAC values: 0.81–4.25 µmol TE mg^−1^ dry weight, superoxide anion scavenging activity values: 27.66–48.92 µmol TE mg^−1^ dry weight) were found in the cultivars. In particular, the cultivars Itrana, Apollo, and Maurino, showed a high amount of total phenols and antioxidant activity, and therefore represent a suitable natural source of biological compounds for use in terms of health benefits.

## 1. Introduction

In the Mediterranean area, the olive (*Olea europaea* L. subsp. *europaea*) is considered to be one of the oldest and important agricultural crops and is characterized by a large number of cultivars used for the production of olive oil and table olives [1,2,3]. While olive oil has been widely studied for its flavor and health benefits, the olive leaf and its chemical composition has only recently attracted interest [4,5].

Olive leaves are a by-product of drupe harvesting and result from the pruning and shaking of olive trees. Olive leaves represent about 10% of the total biomass collected during olive oil production [6], and they are considered to be a cheap raw material which can be used as a useful source of high added-value products (phenolic compounds).

In fact, several studies have investigated the presence of a high number of phenolic compounds in olive leaves such as hydroxytyrosol, rutin, verbascoside, luteolin-7-glucoside, oleuropein, oleuropein aglycone, ligstroside [7], and other compounds such as quinic acid [8]. Generally, oleuropein is the most abundant phenolic compound in olive cultivars [9], which is easily extracted as part of the phenolic fraction of olive fruits, leaves, and seeds, however, it has not been reported in virgin olive oils [7,10].

All these components have been proven to be beneficial in human health because of their antioxidant proprieties. The antihypertensive [11], anticarcinogenic [12], and hypoglycemic, which are antimicrobial activities against *Helicobacter pylori* and *Campylobacter jejuni* [13] have all been demonstrated, as well as the hypocholesterolemic effects of olive leaf extracts [14]. All these positive effects appear to be at least partly related to an antioxidative action [15,16], related mainly to low molecular weight polyphenols such as oleuropein, and polar compounds such as quinic acid.

To date, although quinic acid is well known and characterized in other plant extracts due to its antioxidant potential as an inhibitor of oral pathogens [17,18], it has received little consideration among the olive leaf components.

Biophenols have a wide range of bioactivities [19], and olive leaf extract could be used in cosmetics and pharmaceuticals, and also to improve the shelf-life of foods and to develop functional foods. In fact, olive leaves have been mixed with over-ripened olives to produce oils with a more marked flavor and a higher resistance to oxidation [20], used directly as olive oil supplements [21], and their phenolic extracts have been used to produce dietetic tablets and food supplements [22].

The residues of agricultural and food industries represent a serious problem from an economic and environmental point of view, and thus exploiting such by-products could lead to high value-added products. In this context, our study examined olive leaves from 15 Italian olive cultivars in order to provide basic data on their phenolic composition and antioxidant activities and to predict which one represents the best source of bioactive compounds for functional food, cosmetics, and pharmaceuticals.

The leaves were extracted in ethanol to examine and compare the phenolic profiles, determine the total phenolic content, and quantify the most representative compounds. In addition, the antioxidant activity of the olive leaf extracts was measured using three different complementary assays (DPPH, ORAC, and superoxide anion scavenging activity), to test the potential applications for human use.

## 2. Results and Discussion

### 2.1. Phenolic Compound Analysis

Phenolic compounds were extracted from the leaves of 15 olive cultivars and analyzed using HPLC ESI/MS-TOF, thereby, identifying 26 different compounds. The phenolic compounds identified by negative ionization mode are shown in Table 1, including retention times, experimental and calculated *m*/*z*, molecular formula, errors, score, and literature references.

**Figure 1:**
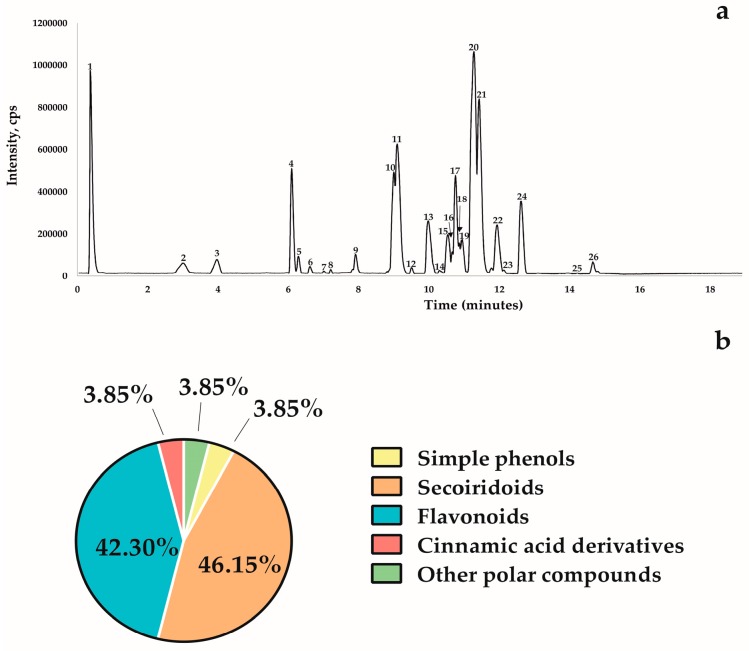
Chromatogram of olive leaves extract and compound classes detected: (**a**) Base peak chromatogram (BPC) of cultivar Itrana olive leaf extract obtained by HPLC ESI/MS-TOF (M − H)^−^: (1) quinic acid, (2) hydroxytyrosol glucoside, (3) secologanoside isomer 1, (4) secologanoside isomer 2, (5) rutin, (6) elenoic acid glucoside, (7) verbascoside, (8) oleuropein aglycone, (9) quercitrin, (10) hydroxyoleuropein, (11) luteolin 7-*O* glucoside isomer 1, (12) luteolin rutinoside, (13) luteolin 7-O glucoside isomer 2, (14) apigenin 7 glucoside, (15) oleuropein diglucoside isomer 1, (16) chrysoerinol 7 glucoside, (17) oleuropein diglucoside isomer 2, (18) oleuropein diglucoside isomer 3, (19) 2-methoxy oleuropein isomer 1, (20) 2-methoxy oleuropein isomer 2, (21) oleuropein; (22) luteolin, (23) quercitin, (24) ligstroside, (25) apigenin 7 glucoside, (26) diosmetin, (**b**) classes of compounds in ethanolic extracts of olive leaves. Detection at 280 nm.

The base peak chromatogram (BPC) profiles did not show a significant qualitative difference between the extracts, indicating that there was no apparent qualitative variation among the phenolic profiles of the olive cultivars analyzed in our study. Figure 1a shows a representative BPC of one of the 15 extracts of the olive leaves belonging to the Itrana cultivar.

The compounds identified correspond to other molecules already reported in olive leaf extracts [23,24,25,26,27,28]. They can be classified into five different chemical classes: polar compounds, simple phenols, secoiridoids, flavonoids, and cinnamic acid derivatives (Figure 1b).

The chemical class of simple phenols and other polar compounds, each representing 3.85% of the total compounds, are represented respectively by hydroxytyrosol glucoside (peak 2, *m*/*z* 315.1095) and quinic acid (peak 1, *m*/*z* 191.0510) (Table 1, Figure 1b).

Twelve secoiridoid molecules (46.15% of the total detected compounds) were identified in the olive leaf extracts of the 15 cultivars: secologanoside isomer 1 (peak 3, *m*/*z* 389.1095), secologanoside isomer 2 (peak 4, *m*/*z* 389.1101), elenoic acid glucoside (peak 6, *m*/*z* 403.1262), oleuropein aglycone (peak 8, *m*/*z* 377.1459), hydroxyoleuropein (peak 10, *m*/*z* 555.1773), oleuropein diglucoside isomers peaks 15, 17, and 18 (at *m*/*z* 701.2307, 701.2306, 701.2291, respectively), 2-methoxy oleuropein isomer 1 and 2 (peak 19, *m*/*z* 569.1898; peak 20, *m*/*z* 569.1899, respectively), oleuropein (peak 21, *m*/*z* 539.1772), and ligstroside (peak 24, *m*/*z* 523.1823) (Table 1; Figure 1b).

Eleven chemical compounds were identified as flavonoids, which represent 42.30% of the total: rutin (peak 5, *m*/*z* 609.1774), quercitrin (peak 9, *m*/*z* 447.0960), luteolin 7-*O* glucoside isomer 1 and 2 (peak 11, *m*/*z* 447.0952; peak 13, *m*/*z* 447.0948, respectively), luteolin rutinoside (peak 12, *m*/*z* 593.1517), apigenin 7 glucoside (peak 14, *m*/*z* 431.0988), chrysoerinol 7 glucoside (peak 16, *m*/*z* 461.1071), luteolin (peak 22, *m*/*z* 285.0419), quercitin (peak 23, *m*/*z* 301.0351), apigenin 7 glucoside (peak 25, *m*/*z* 269.0461) and diosmetin (peak 26, *m*/*z* 299.0566) (Table 1; Figure 1b).

Verbascoside (peak 7, *m*/*z* 623.2013) belongs to the class of cinnamic acid derivatives which represented 3.85% of the total compounds identified (Table 1; Figure 1b).

Quantitative analyses were performed on the most representative components for each of the five chemical classes to which they belonged. All calibration curves of quantified compounds showed a good linearity between peak areas and analyte concentrations, and the regression coefficients were greater than 0.984 in all cases. Detection limits (LODs), quantification limits (LOQs), and other analytic parameters for calibration curve are reported in Table 2.

Table 3 shows the presence of quinic acid, hydroxytyrosol glucoside, luteolin 7-*O* glucoside, 2-methoxy oleuropein, oleuropein, luteolin, and verbascoside in the leaf extracts obtained from the 15 different olive cultivars. The most abundant compounds were quinic acid (about 6–25 g/kg^−1^ DW), luteolin 7-*O* glucoside (about 8–40 g/kg^−1^ DW), oleuropein (about 7–30 g/kg^−1^ DW), 2-methoxy oleuropein (about 2–22 g/kg^−1^ DW), and hydroxytyrosol glucoside (in the range 1–17 g/kg^−1^ DW, except for the cultivar Cellina di Nardò which was about three-fold higher). These results agree with previous studies concerning olive leaves grown in Greece, Tunisia, and Morocco [29,30,31,32].

According to the literature, oleuropein is one of the most abundant compounds in olive leaves [25,33]. Its properties for human health have been widely recognized and include the following: protects the membrane from lipid oxidation and consequently prevents heart disease; has antiviral, cardioprotective and anti-inflammatory properties; improves lipid metabolism; and causes the death of hypertensive cells in cancer patients [6,29,34].

In addition, quinic acid was detected at high concentrations in all the cultivars examined (Table 3). Although quinic acid has been identified in olive leaf extracts, few studies have reported its quantification [8,35]. In other plant species (fruits, vegetables, and commercial derivatives), quinic acid has been quantified because it contributes to their characteristic taste [36,37,38] and has beneficial effects for human health [18]. Conti et al. [17] reported that quinic acid had an antioxidant potential and acts together with other molecules as oral pathogen inhibitors. In addition, quinic acid has been positively associated with symptoms of Pierce disease in the grape variety [39] and in *Olea* during *Xylella fastidiosa* infection [35].

The other two molecules, luteolin (from traces to a maximum of 3 g/kg^−1^ DW) and verbascoside, were detected at low levels in all the cultivars examined, in accordance with the literature [4,26]. Verbascoside is a hydroxycinnamic derivative typical of olive fruit and it has been found in small amounts in olive leaves, as reported by Makowska-Wazs et al. [40] for wild olive trees and by Pereira et al. [41] for the Portuguese olive cultivar Cobrançosa.

**Figure 2:**
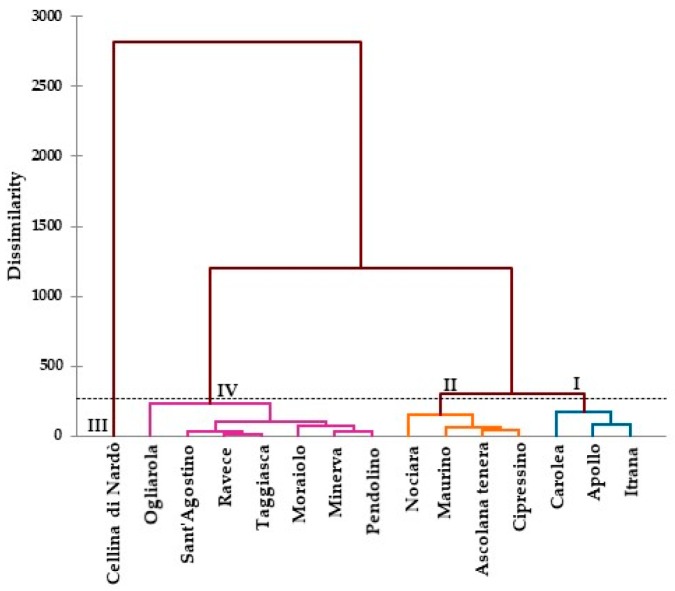
Dendrogram based on the amounts of seven compounds (g/kg^−1^ dry weight), carried out by HPLC ESI/MS-TOF (M − H)^−^, present in the ethanolic extracts of leaves from the 15 Italian olive cultivars.

The cluster analysis based on the amounts of the seven compounds in Table 3 revealed four statistically significant clusters (Figure 2). The olive cultivars attributed to the first cluster were Itrana, Apollo, and Carolea. Leaf samples of this cluster were characterized by higher levels of all the compounds identified and by a high level of quinic acid (25.19, 21.31, and 13.92 g/kg^−1^ DW, respectively) and oleuropein (30.46, 24.48, and 28.30 g/kg^−1^ DW, respectively). Cluster two was distinguished by the mean amounts of luteolin 7-*O* glucoside (values between 27.88 and 35.13 g/kg^−1^ DW) (*p* < 0.05). Cipressino, Ascolana tenera, Maurino and Nociara belong to this cluster. The cultivar Cellina di Nardò represents a third cluster characterized by the highest (*p* < 0.05) amounts of hydroxytyrosol glucoside (57.75 g/kg^−1^ DW). Lastly, Pendolino, Minerva, Moraiolo, Taggiasca, Ravece, Sant’Agostino, and Ogliarola were characterized by the mean values of all compounds quantified.

### 2.2. Antioxidant Activity

Olive trees produce various secondary metabolites to defend themselves against environmental stresses such as high temperatures and UV radiation [6]. The qualitative and quantitative biocompound profile changes depending on the cultivar, phenological stage, maturation degree of the leaf, phytosanitary state, climate, and cultivation area [42,43]. Therefore, olive leaves of the 15 cultivars were collected from trees grown in the same pedoclimatic conditions (same olive orchard, same soil, climate, and culture conditions). As a consequence, the differences found in the phenolic composition and antioxidant activity likely depend, primarily, on the genetic profile of the olive cultivars.

**Figure 3:**
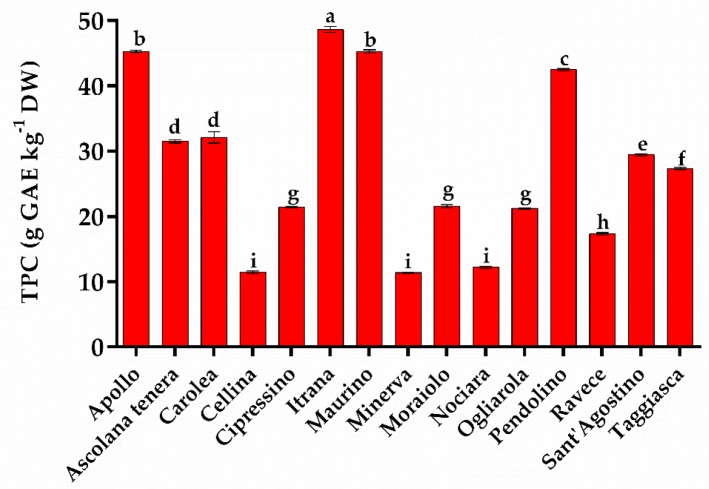
The total phenol content in leaves harvested from the different 15 olive cultivars determined using the Folin–Ciocalteu colorimetric method and expressed as g of gallic acid equivalent per kg^−1^ DW. Data are in triplicate and are presented as mean ± SD. Different letters correspond to statistically different means carried out using ANOVA followed by the Tukey-HSD post hoc test (above the histograms).

Figure 3 shows the total phenol content (TPC) detected in the selected olive cultivars, expressed as g of gallic acid equivalent kg^−1^ dry weight of leaf (g GAE kg^−1^ DW). The TPC in the 15 cultivars ranged between 11 and 49 g GAE kg^−1^ DW (*p* < 0.05).

Our results are generally in line with those reported in the literature for the same Greek [29,33] and Tunisian [32,44] olive cultivars, except for Apollo, Itrana, and Maurino which had considerably higher values. These results were also confirmed by the greater amounts, in these three cultivars, of the individual phenols quantified by HPLC ESI/MS-TOF and reported in Table 3.

The ethanolic leaf extracts were tested using three different in vitro assays (DPPH, ORAC, and superoxide anion scavenging), in order to evaluate the individual antioxidant properties. The tests were chosen because they are an accepted tool for estimating the antioxidant free radical scavenging activities. The DPPH and ORAC assays, had previously been employed on the same matrix [45,46,47].

**Figure 4:**
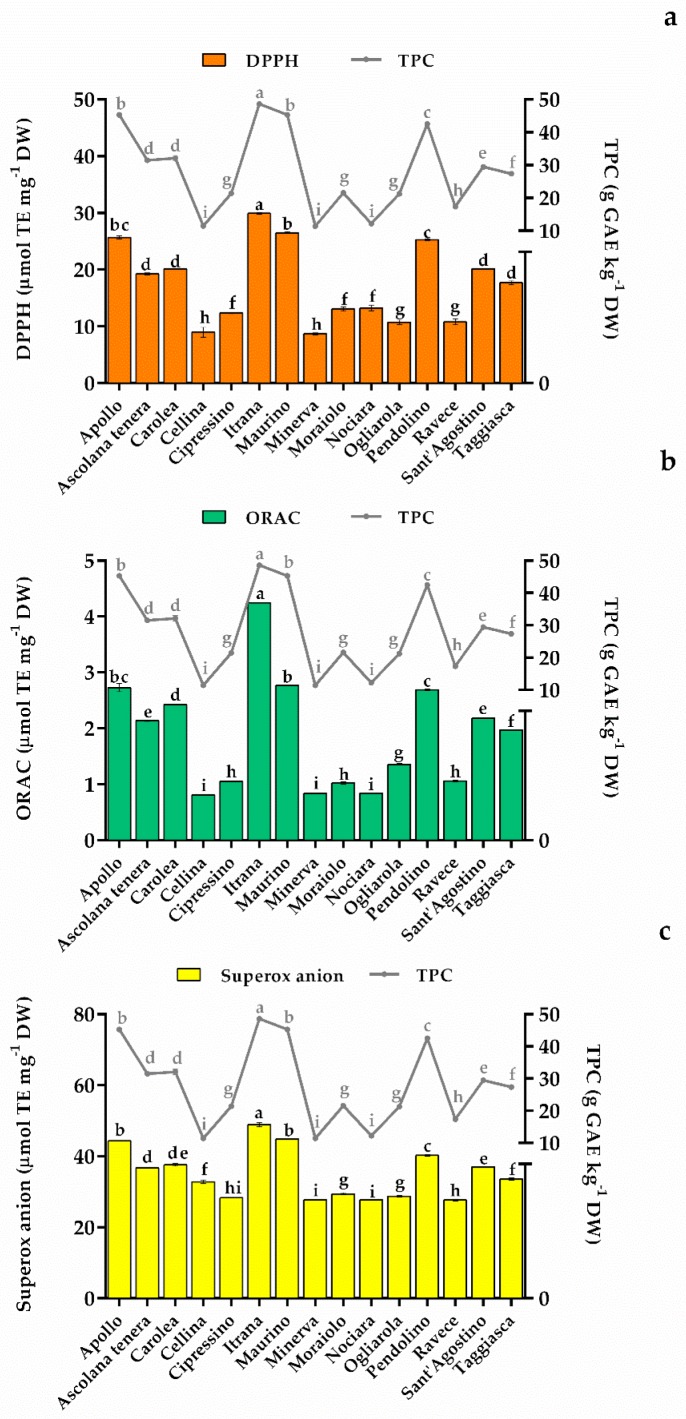
Antioxidant activity of 15 Italian olive leaf extracts evaluated by DPPH (**a**), ORAC (**b**), and superoxide anion scavenging assays (**c**) (results are expressed as µmol Trolox equivalent mg^−1^ of dry weight). Each graph also reports the total phenol content (TPC, expressed as g gallic acid equivalent kg^−1^ dry weight). Statistical analysis was performed by ANOVA followed by the Tukey-HSD post hoc test. Different letters correspond to statistically different means.

The results of all assays, expressed as µmol Trolox equivalent mg^−1^ dry weight (µmol TE mg^−1^ DW), are shown in Figure 4. According to the data in the literature [6,46,48], the three assays showed good antioxidant activity for all the olive leaf ethanolic extracts, indicating statistically significant differences among the cultivars analyzed (*p* < 0.05).

Itrana, Apollo, and Maurino cultivars showed the greatest antioxidant activity in all three tests, and significantly correlated with the total phenol content (Figure 4) and with the high amounts of quinic acid, oleuropein, and luteolin 7-*O* glucoside (Table 3). In fact, a high phenolic content in extracts is generally a good indicator of the antioxidant properties because there is a direct relationship between the phytochemical content and antioxidant activity [49]. As shown in Figure 4, a close correlation was found between the total phenol content and the antioxidant activity of all the cultivars. This is due to the high number of phenolic components and their strong scavenging activity [42,50]. The data also highlight the importance of the synergistic activity of the bioactive compounds in the extracts, which is often more beneficial than an individually isolated constituent [6].

Figure 4 shows that the DPPH assay values ranged from 8.67 (Minerva) to 29.89 (Itrana) µmol TE mg^−1^ DW, the ORAC assay values varied from 0.81 (Cellina di Nardò) to 4.25 (Itrana) µmol TE mg^−1^ DW, and, lastly, the superoxide anion scavenging values ranged from 27.66 (Minerva) to 48.92 (Itrana) µmol TE mg^−1^ DW. Of the various parts of the olive tree, the olive leaves have the highest antioxidant and scavenging ability [48], however, it is difficult to compare antioxidant activity results with the literature because of the heterogeneity both in the sample preparation, and the tests and data expressions. However, albeit with some variations, the high values of antioxidant activity obtained through DPPH, ORAC, and anion superoxide scavenging assays are in agreement with the values reported for olive by-products by Orak et al. [5], Xie et al. [46], and Ciriminna et al. [51].

The data obtained concerning the biophenol composition and the antioxidant activity of the olive leaf extract appear encouraging for further potential uses of olive leaves [6].

## 3. Materials and Methods

### 3.1. Plant Material and Sample Preparation

The leaves of O. europaea were collected in October 2018 from 15 cultivars (Apollo, Ascolana tenera, Carolea, Cellina di Nardò, Cipressino, Itrana, Maurino, Minerva, Moraiolo, Nociara, Ogliarola, Pendolino, Ravece, Sant’Agostino, and Taggiasca). The trees of each cultivar were of the same age (about 10 years old), grown in the same agronomical and environmental conditions, and were negative for the most common olive pathogens [52,53]. The selected cultivars were among the most widespread and representative of the Italian oil and table olive germplasm (Table 4).

The leaf samples were collected from different parts of three trees for the cultivars and subsequently pooled into a single cultivar sample. The leaves were ground with a mortar and pestle in liquid nitrogen to which the ethanol solution at 60% (1:10) was added, and they were left to stir in the dark for 2 h. After centrifugation at a maximum speed (5000× *g*), the resulting solutions were filtered into glass vials using a 0.2 μm PFTE membrane and analyzed as described below. Three replicates for each harvested sample were carried out.

### 3.2. HPLC ESI/MS-TOF Analysis of Leaf Extracts

The phenolic characterization and quantification were performed using an Agilent 1200 liquid chromatography system (Agilent Technologies, Palo Alto, CA, USA) equipped with a standard autosampler and analytical column Agilent Zorbax extended C18 (5 × 2.1 cm, 1.8 µm), as reported by Nicolì et al. [54] and Vergine et al. [55]. The HPLC system was coupled to an Agilent diode-array detector. The detection wavelength was 280 nm and an Agilent 6320 TOF mass spectrometer was equipped with a dual ESI interface (Agilent Technologies) operating in a negative ion mode. Detection was carried out within a mass range of 50–1700 *m*/*z*. Accurate mass measurements of each peak from the total ion chromatograms (TICs) were obtained by using an ISO pump (Agilent G1310B) using a dual nebulizer ESI source that introduced a low flow (20 μL min^−1^) of a calibration solution containing the internal reference masses at *m*/*z* 112.9856, 301.9981, 601.9790, 1033.9881, in negative ion mode. The accurate mass data of the molecular ions were processed using Mass Hunter software (Agilent Technologies).

The compounds were quantified using calibration curves of authentic standards (quinic acid, hydroxytyrosol, oleuropein, luteolin 7-O glucoside, luteolin, and verbascoside) and the regression equation and the correlation coefficient (r^2^) were calculated, as reported by Luvisi et al. [35].

### 3.3. Total Phenol Content (TPC) and Antioxidant Activity

The total phenol content was determined using the spectrophotometric Folin-Ciocolteau method [56]. Data were expressed as g of gallic acid equivalent kg^−1^ of Dry Weight (DW).

Antioxidant activity was evaluated using different assays: the DPPH test was carried out as reported by Bondet et al. [57]; ORAC test, as reported by Ou et al. [58]; superoxide anion scavenging was also analyzed as described by Dasgupta et al. [59]. All the assays were performed in triplicate. The antioxidant activities were expressed as µmol of Trolox equivalent mg^−1^ of dry weight (DW).

### 3.4. Statistical Analysis

The results were subjected to one-way ANOVA analysis, followed by the Tukey-HSD (honestly significant difference) post hoc test (*p* < 0.05). All data were reported as the mean ± SD with at least three replications for each olive leaf sample. Statistical analyses were performed using GraphPad version 6.01(GraphPad Software, San Diego, CA, USA).

Data from the quantitative analyses of seven compounds were also used for the hierarchical cluster analysis using Euclidean distances. Computations were performed using XLSTAT version 18.07.01. (Addinsoft Inc., Long Island City, NY, USA).

## 4. Conclusions

Olive leaves are considered as by-products of the olive tree cultivation and oil industry, however, in recent years, interest in the alternative uses of these agro-food by-products has increased considerably. In view of the large quantity of this “by-product” available in Italy, we analyzed the bioactive components and the antioxidant activity of leaves belonging to 15 Italian olive cultivars. The data obtained showed a high content in total phenols and a high antioxidant activity for all olive leaf extracts. Among the cultivars analyzed, three (Itrana, Maurino and Apollo) showed the highest content of phenolic compounds which correlated with the highest antioxidant activity.

Therefore, olive leaves collected from all the tested Italian cultivars represent an important and inexpensive natural source of antioxidants for use in various applications and in products with potentially beneficial effects on human health.

## Figures and Tables

**Table 1:** List of compounds extracted from olive leaves of 15 cultivars and identified by HPLC ESI/MS-TOF.

Compound	RT (min) ^a^	(M−H)^−^	*m*/*z* Exp ^b^	*m*/*z* Clc ^c^	Diff. (ppm) ^d^	Score ^e^	Ref.
*Quinic acid	0.365	C_7_H_11_O_6_	191.0510	191.0561	−5.89	90.44	[23,24,25]
*Hydroxytyrosol glucoside	2.965	C_14_H_19_O_8_	315.1095	315.1085	−1.26	96.62	[23,24,25]
Secologanoside isomer 1	3.960	C_16_H_21_O_11_	389.1095	389.1089	−1.11	88.91	[23,24,25]
Secologanoside isomer 2	6.116	C_16_H_21_O_11_	389.1101	389.1089	−2.62	96.13	[24,26]
*Rutin	6.230	C_27_H_29_O_16_	609.1474	609.1461	−2.15	90.20	[24,26]
Elenoic acid glucoside	6.630	C_17_H_23_O_11_	403.1262	403.1246	−3.68	80.90	[24,26]
*Verbascoside	6.950	C_29_H_35_O_15_	623.2013	623.1618	−0.05	93.73	[26,27]
Oleuropein aglycone	7.194	C_16_H_25_O_10_	377.1459	377.1453	−1.23	92.94	[24]
*Quercitrin	7.944	C_21_H_19_O1_1_	447.0960	447.0933	−6.05	89.44	[27]
Hydroxyoleuropein	9.036	C_25_H_31_O_14_	555.1773	556.1803	−2.04	97.55	[24,27]
*Luteolin 7-O glucoside isomer 1	9.119	C_21_H_19_O_11_	447.0952	447.0933	−3.93	77.64	[24,25]
*Luteolin rutinoside	9.517	C_27_H_29_O_15_	593.1517	593.1512	−0.87	97.79	[25]
*Luteolin 7-O glucoside isomer 2	9.998	C_21_H_19_O_11_	447.0948	447.0933	−3.03	96.13	[23,24,25]
Apigenin 7 glucoside	10.010	C_21_H_19_O_10_	431.0988	431.0984	−0.79	97.82	[23,24,25]
Oleuropein diglucoside isomer 1	10.545	C_31_H_41_O_8_	701.2307	701.2298	−0.60	93.83	[23,24,25]
Chrysoerinol 7 glucoside	10.650	C_22_H_21_O_11_	461.1071	461.1089	4.06	79.09	[23]
Oleuropein diglucoside isomer 2	10.728	C_31_H_41_O_8_	701.2306	701.2298	−0.49	94.85	[23,24,25]
Oleuropein diglucoside isomer 3	10.893	C_31_H_41_O_8_	701.2291	701.2298	3.20	98.67	[23,24,25]
2-methoxy oleuropein isomer 1	11.175	C_26_H_33_O_14_	569.1898	569.1876	−3.76	85.77	[25]
2-methoxy oleuropein isomer 2	11.258	C_26_H_32_O_14_	569.1899	569.1876	−3.64	97.16	[25]
*Oleuropein	11.406	C_15_H_9_O_13_	539.1772	539.1770	0.03	97.14	[23,24,25,27]
*Luteolin	11.939	C_15_H_9_O_6_	285.0419	285.0405	−4.87	97.08	[23,24,25,27]
*Quercetin	12.036	C_15_H_9_O_7_	301.0351	301.0354	1.10	96.04	[24,25,28]
Ligstroside	12.611	C_25_H_31_O_12_	523.1823	523.1821	−0.03	97.55	[26]
*Apigenin 7 glucoside	14.263	C_15_H_9_O_5_	269.0461	269.0455	−1.77	98.70	[23]
Diosmetin	14.694	C_16_H_11_O_6_	299.0566	299.0561	−1.43	98.50	[23]

**^a^** Retention time, **^b^** *m*/*z* experimental, **^c^** *m*/*z* calculated, **^d^** difference between the observed mass and the theoretical mass of the compound (ppm), **^e^** isotopic abundance distribution match: a measure of the probability that the distribution of isotope abundance ratios calculated for the formula matches the measured data. * Confirmed by authentic chemical standards.

**Table 2:** Parameters of calibration curves, limits of detection (LODs), limits of quantification (LOQs) and relative standard deviation (RSD) for the HPLC method validation of phenolic assays in ethanolic olive leaf extract.

Standard Compound	Slope	Intercept	r^2^	LOD (µg mL^−^^1^)	LOQ (µg mL^−^^1^)	RSD (%)
Quinic acid	2.19 × 10^5^	−7.17 × 10^4^	0.999	1.81	6.03	0.82
Hydroxytyrosol	1.95 × 10^5^	−2.57 × 10^5^	0.999	2.60	8.66	0.78
Luteolin 7-O glucoside	1.37 × 10^6^	1.53 × 10^6^	0.988	1.25	4.15	0.77
Oleuropein	1.81 × 10^6^	5.01 × 10^5^	0.997	0.77	2.56	0.81
Luteolin	1.61 × 10^6^	3.91 × 10^6^	0.989	0.13	0.45	0.74
Verbascoside	2.60 × 10^5^	3.08 × 10^5^	0.984	0.15	0.50	0.87

**Table 3:** Quantitative analysis of seven compounds (g/kg^−1^ dry weight), carried out by HPLC ESI/MS-TOF (M-H)^−^, of the ethanolic leaf extracts of 15 olive cultivars. Different letters correspond to statistically different means carried out using ANOVA followed by the Tukey-HSD post hoc test. All the data are triplicate and are presented as mean ± SD.

Cultivar	Quinic Acid	Hydroxytyrosol Glucoside	Luteolin 7-*O* Glucoside	2-Methoxy Oleuropein *	Oleuropein	Luteolin	Verbascoside
**Apollo**	21.31 ± 0.29**b**	8.17 ± 0.07**e**	39.78 ± 0.03**a**	10.51 ± 0.15**c**	24.48 ± 0.07**d**	2.66 ± 0.10**b**	0.16 ± 0.02**cd**
**Ascolanatenera**	12.71 ± 0.04**h**	10.96 ± 0.05**d**	32.75 ± 0.08**c**	7.80 ± 0.05**g**	22.06 ± 0.08**f**	0.15 ± 0.02**f**	0.18 ± 0.04**bc**
**Carolea**	13.93 ± 0.01**e**	17.34 ± 0.10**b**	35.05 ± 0.06**b**	12.71 ± 0.03**b**	28.30 ± 0.07**b**	0.10 ± 0.01**f**	0.13 ± 0.01**defg**
**Cellina di** **Nardò**	11.25 ± 0.07**i**	57.75 ± 0.11**a**	23.31 ± 0.22**g**	22.14 ± 0.09**a**	9.69 ± 0.02**p**	2.62 ± 0.0**b**	0.20 ± 0.04**ab**
**Cipressino**	13.31 ± 0.06**f**	3.58 ± 0.01i**l**	29.13 ± 0.07**e**	9.42 ± 0.05**d**	25.52 ± 0.03**c**	0.21 ± 0.01**f**	0.22 ± 0.05**a**
**Itrana**	25.19 ± 0.04**a**	1.13 ± 0.02**q**	31.56 ± 0.09**d**	8.42 ± 0.14**f**	30.46 ± 0.12**a**	1.54 ± 0.0**c**	0.11 ± 0.01**fg**
**Maurino**	14.81 ± 0.03**d**	2.05 ± 0.05**o**	27.88 ± 0.10**f**	4.08 ±0.07**m**	18.53 ± 0.07**h**	3.02 ± 0.0**a**	0.10 ± 0.02**g**
**Minerva**	6.05 ± 0.02**n**	2.42 ± 0.03**n**	15.95 ± 0.05**n**	3.32 ± 0.10**o**	17.38 ± 0.17**l**	1.06 ± 0.0**de**	0.18 ± 0.02**bc**
**Moraiolo**	9.20 ± 0.07**m**	11.88 ± 0.08**c**	20.12 ± 0.02**i**	5.56 ± 0.04**h**	14.61 ±0.01**m**	1.41 ±0.03**cd**	0.14 ± 0.04**def**
**Nociara**	10.22 ± 0.02**l**	7.14 ± 0.02**g**	35.13 ± 0.10**b**	3.92 ± 0.05**n**	9.89 ± 0.10**o**	0.18 ± 0.01**f**	0.10 ± 0.02**g**
**Ogliarola**	6.24 ± 0.07**n**	7.90 ± 0.01**f**	8.69 ± 0.16**o**	8.82 ± 0.02**e**	7.49 ± 0.04**q**	0.21 ± 0.01**f**	0.14 ± 0.02**def**
**Pendolino**	12.55 ± 0.06**h**	1.69 ± 0.15**p**	17.84 ± 0.04**m**	2.55 ± 0.05**q**	12.58 ± 0.09**n**	0.88 ± 0.02**e**	0.15 ± 0.02**cde**
**Ravece**	13.02 ± 0.01**g**	3.72 ± 0.04**i**	15.85 ± 0.06**n**	3.07 ± 0.08**p**	18.12 ± 0.03**i**	0.09 ± 0.01**f**	0.13±0.01**defg**
**Sant Agostino**	16.50 ± 0.02**c**	3.48 ± 0.01**m**	21.57 ± 0.03**h**	5.28 ± 0.01**i**	23.55 ± 0.03**e**	0.16 ± 0.01**f**	0.11 ± 0.01**fg**
**Taggiasca**	12.54 ± 0.02**h**	4.58 ± 0.07**h**	18.14 ± 0.09**l**	4.14 ± 0.02**l**	21.74 ± 0.05**g**	0.95 ± 0.01**e**	0.12 ± 0.02**efg**

* 2-methoxy oleuropein was quantified using the oleuropein standard.

**Table 4:** List of *Olea europaea* L. cultivars analyzed, their attitude and principal area of cultivation.

Cultivars	Attitude	Principal Area of Cultivation
**Apollo**	olive oil	Tuscany (Central Italy)
**Ascolana tenera**	oil and table olive	Abruzzo (Southern Italy)
**Carolea**	oil and table olive	Calabria, Basilicata (Southern Italy)
**Cellina di Nardò**	oil and table olive	Apulia (Southern Italy)
**Cipressino**	olive oil	Apulia, Sardinia (Southern Italy)
**Itrana**	oil and table olive	Lazio (Central Italy)
**Maurino**	olive oil and pollinator	Tuscany (Central Italy)
**Minerva**	olive oil	Tuscany (Central Italy)
**Moraiolo**	olive oil	Tuscany, Umbria (Central Italy)
**Nociara**	olive oil	Apulia (Southern Italy)
**Ogliarola**	olive oil	Apulia (Southern Italy)
**Pendolino**	olive oil and pollinator	Tuscany (Central Italy)
**Ravece**	oil and table olive	Campania (Southern Italy)
**Sant’Agostino**	oil and table olive	Apulia (Southern Italy)
**Taggiasca**	oil and table olive	Liguria (Northern Italy)
